# Brain-inspired wiring economics for artificial neural networks

**DOI:** 10.1093/pnasnexus/pgae580

**Published:** 2025-01-07

**Authors:** Xin-Jie Zhang, Jack Murdoch Moore, Ting-Ting Gao, Xiaozhu Zhang, Gang Yan

**Affiliations:** School of Physical Science and Engineering, Tongji University, Shanghai 200092, P. R. China; National Key Laboratory of Autonomous Intelligent Unmanned Systems, MOE Frontiers Science Center for Intelligent Autonomous Systems, Tongji University, Shanghai 200092, P. R. China; School of Physical Science and Engineering, Tongji University, Shanghai 200092, P. R. China; National Key Laboratory of Autonomous Intelligent Unmanned Systems, MOE Frontiers Science Center for Intelligent Autonomous Systems, Tongji University, Shanghai 200092, P. R. China; School of Physical Science and Engineering, Tongji University, Shanghai 200092, P. R. China; National Key Laboratory of Autonomous Intelligent Unmanned Systems, MOE Frontiers Science Center for Intelligent Autonomous Systems, Tongji University, Shanghai 200092, P. R. China; School of Physical Science and Engineering, Tongji University, Shanghai 200092, P. R. China; National Key Laboratory of Autonomous Intelligent Unmanned Systems, MOE Frontiers Science Center for Intelligent Autonomous Systems, Tongji University, Shanghai 200092, P. R. China; Chair for Network Dynamics, Center for Advancing Electronics Dresden (cfaed) and Institute for Theoretical Physics, Technical University of Dresden, Dresden 01062, Germany; School of Physical Science and Engineering, Tongji University, Shanghai 200092, P. R. China; National Key Laboratory of Autonomous Intelligent Unmanned Systems, MOE Frontiers Science Center for Intelligent Autonomous Systems, Tongji University, Shanghai 200092, P. R. China; CAS Center for Excellence in Brain Science and Intelligence Technology, Chinese Academy of Sciences, Shanghai 200031, P. R. China

**Keywords:** wiring cost, brain-like computing, interpretable AI, bio-inspired AI, sparse neural networks

## Abstract

Wiring patterns of brain networks embody a trade-off between information transmission, geometric constraints, and metabolic cost, all of which must be balanced to meet functional needs. Geometry and wiring economy are crucial in the development of brains, but their impact on artificial neural networks (ANNs) remains little understood. Here, we adopt a wiring cost-controlled training framework that simultaneously optimizes wiring efficiency and task performance during structural evolution of sparse ANNs whose nodes are located at arbitrary but fixed positions. We show that wiring cost control improves performance across a wide range of tasks, ANN architectures and training methods, and can promote task-specific structural modules. An optimal wiring cost range provides both enhanced predictive performance and high values of topological properties, such as modularity and clustering, which are observed in real brain networks and known to improve robustness, interpretability, and performance of ANNs. In addition, ANNs trained using wiring cost can emulate the connection distance distribution observed in the brains of real organisms (such as *Ciona intestinalis* and *Caenorhabditis elegans*), especially when achieving high task performance, offering insights into biological organizing principles. Our results shed light on the relationship between topology and task specialization of ANNs trained within biophysical constraints, and their geometric resemblance to real neuronal-level brain maps.

**Significance Statement**

Many important advances in AI have been inspired by the brain. Wiring cost, which is the metabolic and material expense of maintaining synaptic connections and increases with the distances spanned by neuronal connections as well as their cross-sections, is fundamental to the development of brain structure. Previous work showed that incorporating into artificial neural networks a notion of wiring cost which represents both connection weights and distances leads to modular structures which enhance interpretability. We show that the method can also enhance task performance, and that both performance and interpretability advantages extend across a broad range of tasks, network models, and training conditions. The framework can even replicate geometrical features observed in biological neural networks.

## Introduction

The strong impact of wiring patterns on the performance of artificial neural networks (ANNs) is becoming increasingly recognized. Network science and graph theory have been employed to analyze ANNs (1–3), revealing that wiring patterns play a critical role in their functionality. For example, deep neural networks wired with small-world topology have shown competitive performance in image recognition (4) and neural networks with modular structures have been found to mitigate catastrophic forgetting during multitask learning (5–7). Higher clustering coefficient has also been identified as beneficial to network performance (8). Furthermore, researchers have observed the emergence of modularity (9), scale-free topology (10), and structurally balanced patterns in sparsified neural networks (11). These insights have significantly contributed to our understanding of ANN design and will continue to shape future research in this field.

While ANNs inspired by brain-like wiring patterns have achieved breakthroughs in autonomous driving (12), computer vision (13, 14), and natural language processing (15), significant differences still exist between artificial and biological neural systems. Biological brain networks are physically embedded in 3D space, and the establishment and maintenance of axonal connections incurs physical cost known as wiring cost, typically approximated as the total volume of connective material, the sum of products of Euclidean lengths and cross-sectional areas of interneuronal connections, or simply the sum of these lengths (16). Extensive evidence indicates that the organization of brain networks represents a trade-off between physical cost and topological value (17). Notably, the mammalian cerebral cortex (18) and the nervous system of *Caenorhabditis elegans* (16) have been observed to minimize costs associated with interconnecting wiring. The spatial dependence of synaptic connection probabilities in biological networks, as seen in the mammalian neocortex (19) and the cellular nervous system of *C. elegans*, has also been attributed to wiring cost control (20). Recognition of the volume constraints in biological brains inspired the use of total wiring length as a mathematically advantageous and technologically meaningful measure of complexity in electronic circuits and also led to the development of asymptotically optimally efficient circuits for performing fundamental tasks in computer vision (21–23). Also, exerting downward pressure on wiring cost while training neural networks has been shown to provide more interpretable structures (5, 6, 24–31), improved generalization (24, 26), faster training (24, 28), and higher performance in a range of tasks (5, 26, 27). However, in most cases the wiring of ANNs is primarily driven by engineering objectives and computational requirements, often leading to higher wiring cost (32). Although the concept of wiring economy has been extensively explored in biological neural networks, its application in the realm of ANNs has been limited, especially with regard to formulations which accommodate connective diversity by incorporating connection weight alongside connection distance (but see Refs. (24, 29, 31) for exceptions).

To address this gap and shed light on the role of geometry in ANNs, we adopt a cost-controlled framework (31) and systematically investigate how wiring cost constraints which represent both the distances and weights of connections affect the correlation between topology, task specialization of ANNs, and their emulation of real biological neural networks. By spatially embedding the components (neurons) of ANNs in arbitrary but fixed locations within geometric structures, we aim to optimize the topological connectivity of models while minimizing wiring cost. Our results show that considering biophysical constraints, including geometry and wiring cost, enhances the performance of various recurrent network models and reveals a nonmonotonic relationship between task performance and wiring cost entailing an optimal region where models maintain high performance and exhibit beneficial structural properties alongside minimal wiring costs. Additionally, we introduce simple yet effective measures to quantify the relationship between self-organizing topological modules formed by networks’ units and task representations, showing that structural modularity corresponds to task specialization. Finally, by considering the model organisms *Ciona intestinalis* and *C. elegans*, we find that the model trained with biophysical constraints can achieve high performance while recreating the distance distribution observed in biological neural networks, yielding insights into the organization of biological networks. This utilization of real biological circuits distinguishes our study from previous related work (5, 6, 24–31). We also go beyond our study’s most similar predecessor (31) by demonstrating that the considered method of incorporating distances and weights into training can improve task performance as well as interpretability, and by considering many more combinations of network architectures, training strategies, pruning strategies, tasks, and sparsities.

## Results

### Wiring cost-controlled training framework

**Fig. 1.**
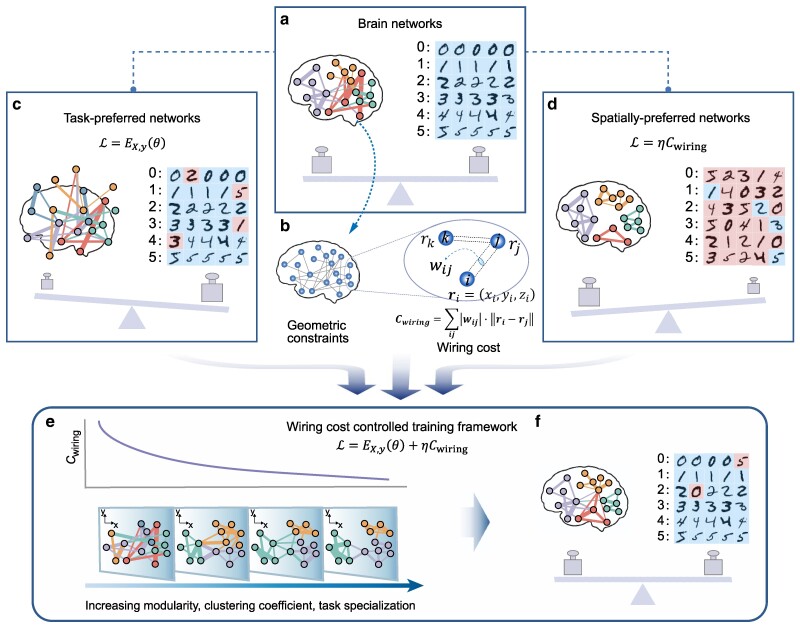
Wiring cost-controlled training framework. The proposed wiring cost-controlled training framework unites features from task-preferred and spatially preferred networks which are crucial in brain networks. a) Brain networks must achieve high performance in the tasks necessary for an organism’s survival while balancing network metabolic/wiring cost and satisfying geometric constraints. b) The wiring cost $C_{\mathrm{wiring}}$ can be approximated as the volume of interconnected neurons, calculated by summing the Euclidean distances ($r_{i}=(x_{i},y_{i},z_{i})$ represents the position of neuron *i*) between all neurons multiplied by the strength of interneuronal connections ($w_{ij}$). Observed brain networks have inspired both task-preferred and spatially preferred networks. c) Task-preferred network, trained using the cost function $L=E_{X,y}(\theta )$ to perform tasks without consideration for geometry and wiring cost. d) Spatially preferred network that can be trained using cost function $L=\eta C_{\mathrm{wiring}}$ to optimize wiring cost and adhere to geometric constraints, but lacks reliability in performing machine learning tasks. e, f) We combine task-preferred and spatially preferred networks via the cost function $L=E_{X,y}(\theta )+\eta C_{\mathrm{wiring}}$ (31). By employing sparsification strategies, we optimize the weights and topology of spare networks through iterative pruning and regrowth of connections during training. This framework facilitates networks that emulate real-world brain networks by (e, top) minimizing wiring cost $C_{\mathrm{wiring}}$, while (e, bottom) enhancing modularity, clustering, and task specialization, (f) achieving high task performance alongside low wiring cost while respecting geometric constraints. Here, we illustrate task performance using the classic MNIST handwritten numeral classification task, blue (red) squares are used to indicate correctly (incorrectly) recognized digits. We employ a weight icon to visually represent the extent of achievement of wiring efficiency and task performance goals, and a scale to signify the trade-off between these goals.

The brain is a spatially embedded complex network formed by the interconnection of neurons through synapses. Biological pressure works to minimize physical wiring costs incurred by the establishment and maintenance of synaptic connections, while maximizing topological properties, such as modularity and clustering, critical for brain function (33, 34). In order to achieve this delicate balance, the brain carefully trades off the requirements of high-performance task execution required for organism survival, geometric constraints, and the management of network metabolism/wiring cost (Fig. 1a and b). In contrast, AI systems, such as ANNs, belong to the category of task-preferred networks driven by computing requirements, with wiring patterns which lack inherent geometric or cost constraints (Fig. 1c). Conversely, there are spatially preferred networks, such as Internet routers and autonomous systems (35), that strictly adhere to geometric constraints and aim to minimize wiring cost, but which are not designed to perform machine-learning tasks (Fig. 1d). This leads to the question of whether ANNs can be trained to achieve a harmonious balance between task performance and wiring efficiency, akin to that in the brain. To explore this intriguing possibility, we adopt a wiring cost-controlled training framework for sparse ANNs through incorporating geometric constraints entailed by the network’s spatial realization (31) (Fig. 1e and f).

We commence the process with spatially embedded sparse neural networks (Fig. 1e bottom). Sparse neural networks have demonstrated the ability to achieve comparable performance to fully connected networks while utilizing fewer connections (trainable parameters) (36, 37), which aligns with the sparse topology of biological neural networks (38). During the training process, we optimize both the parameters (weights) and sparse topology of the neural networks while strictly maintaining the total number of connections. By incorporating biophysical constraints that minimize wiring cost (31), defined as the sum of the interneuronal distances (Fig. 1b), the network is empowered to enhance modularity and clustering within its structure (Fig. 1e). This optimization not only promotes functional task specialization but also facilitates efficient trade-offs between network structure and functionality (Fig. 1f).

To achieve this, our training strategy encompasses two key aspects. The first goal is to search for the optimal configuration within the parameter space θ in order to reduce the error function $E_{X,y}(\theta )$ given a set of input features ***X*** and target outputs ***y***. To establish a unified objective function that takes into account both task performance and wiring efficiency, simultaneously we add an additional regularization term to the loss function L according to


(1) $$L=E_{X,y}(\theta )+\eta C_{\mathrm{wiring}},$$

where $C_{\mathrm{wiring}}$ is the wiring cost function, which can be approximated by quantifying the physical wiring volume (Fig. 1b, also see Methods for details), and *η* controls the penalty of wiring cost (31). The second intention is that we encourage the formation of short-distance connections instead of long-distance connections. This is achieved via dynamic sparse rewiring strategies (10, 39, 40), which effectively optimize the topology of sparse neural networks by iteratively pruning and rewiring connections between neurons. Specifically, in the rewiring phase, we introduce into the dynamic sparse rewiring algorithms a probability for new connections inversely proportional to the distance the connection would span.

### Trade-offs between wiring efficiency, task performance, and topology properties

To investigate the impact of geometric and wiring cost constraints on the performance of sparse neural networks, we evaluate our wiring cost-controlled training framework across four classes of recurrent networks, comprising recurrent neural networks (RNNs), continuous-time RNNs (CT-RNNs) (41), neural ordinary differential equation networks (Neural ODEs) (42), and gated recurrent units (GRUs) (43), see Methods for more details. Each model shares a common architecture featuring a single sparse hidden layer with 131 recurrent units, where the connection density among recurrent units is constrained to be less than 10%. The input features are fed to each recurrent unit through a learnable linear layer, and the output of the recurrent layer is projected to the output neurons via another learnable linear layer. To imbue the recurrent network with spatial geometry, all recurrent units in the hidden layer are treated as independent nodes and are randomly assigned locations on the circumference (see Supplementary Information, Section I).

**Fig. 2.**
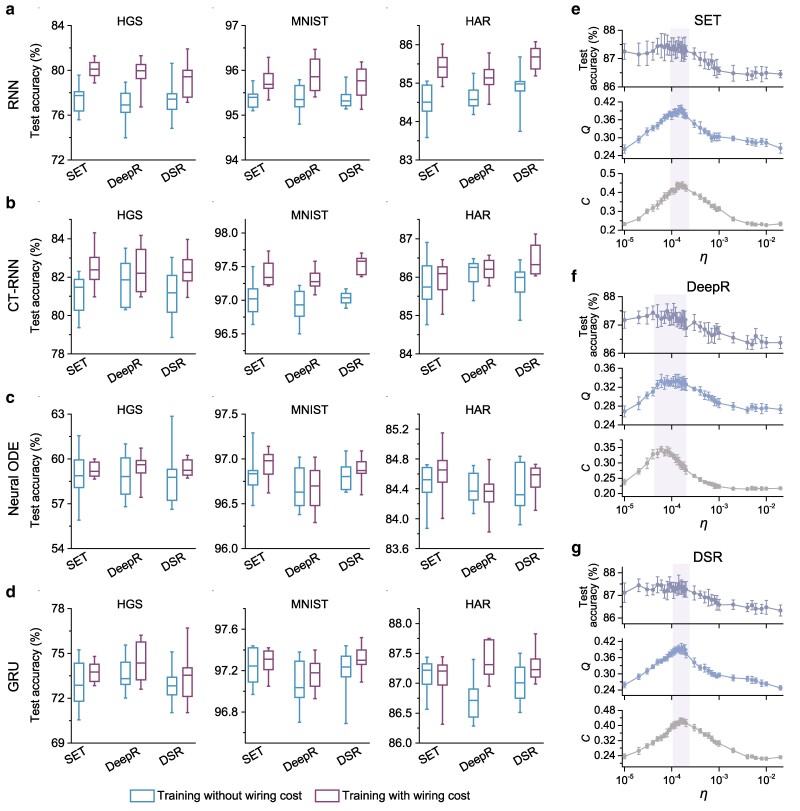
The trade-off between network performance, topological characteristics, and cost control. a–d) Simultaneous pursuit of high network performance and low wiring cost. We evaluate the accuracy of RNN (a), CT-RNN (b), neural ODE (c), and GRU (d) models trained on HGS, MNIST, and HAR datasets. Each model comprises a sparse hidden layer with 131 neurons, limiting the probability of connections between neurons in this layer to no more than 10%. The performance comparison between models trained without (blue) and with (purple) wiring cost control, based on dynamic sparse rewiring strategies: SET, DeepR, and DSR. The boxplots present the minimum, maximum, median, and interquartile ranges (spanning from the 25th to the 75th percentile). e–g) Variations in test accuracy (Top) of sparse GRUs trained on the HAR dataset, and modularity (*Q*, middle) and average clustering coefficient (*C*, bottom) of the GRU hidden layer, are showcased as functions of *η*. The shaded intervals correspond to the optimal region of wiring cost, where sparse GRUs maintain high task performance while having high modularity and clustering. Error bars represent the standard deviation over ten independent runs. The hyperparameters used in these experiments are listed in Supplementary Information Table S1.

For each model, we adopt our wiring cost-controlled training framework based on three dynamic sparse rewiring algorithms, specifically, sparse evolutionary training (SET) (10), deep rewiring (DeepR) (39), and dynamic sparse reparameterization (DSR) (40), as detailed in Methods. We set as the baseline the performance of models trained without considering wiring cost. Implementing experiments on the hand gesture segmentation (HGS), MNIST, and human activity recognition (HAR) datasets (see Methods for more details), we observe enhanced test accuracy in models trained using the wiring cost-controlled framework compared to those trained without wiring cost considerations (Fig. 2a–d). This trend persists across different dynamic sparse rewiring algorithms, network models, network sparsities (see Supplementary Information, Section II), and datasets (including naturalistic task datasets (44, 45), see Supplementary Information Section III), indicating that embedding the wiring cost constraint supports and even improves high-performing models. Considering wiring cost constraints can also improve upon either $L_{1}$ (46) or $L_{2}$ (47) regularization (see Supplementary Information Sections IV and V for supporting statistical analysis using constrained surrogates (48)).

So far, our findings have highlighted the beneficial impact of geometric and wiring cost constraints on enhancing network performance. We are also interested in the relationship between wiring cost, network performance, and topology of sparse neural networks. To unravel these dynamics, we conduct a systematic analysis of task prediction accuracy and structural properties of models’ wiring patterns (Fig. 2e–g). Here, as in several other experiments, we take the sparse GRU as an example, but we would expect similar patterns for other models. We consider varying the wiring cost penalty coefficient *η* across several orders of magnitude, ranging from $10^{-5}$ to $10^{-2}$, while using the SET, DeepR, and DSR rewiring algorithms to train sparse GRUs for the HAR task. As *η* increases from a low value, test accuracy can initially increase before plateauing and remaining consistently high within a certain range. However, as *η* is increased beyond this interval, performance decreases (Fig. 2e–g top).

Furthermore, to analyze the topology of these optimized GRUs from the perspective of network science, we first transform the wiring pattern of the hidden layer’s recurrent units into an undirected graph by mapping each recurrent unit to a single vertex and the interconnections between these units to edges. We focus on two fundamental topological characteristics commonly observed in empirical brain networks: modularity (Fig. 2e–g middle) and clustering (Fig. 2e–g bottom). We find that both modularity *Q* and average clustering coefficient *C* (see Methods for more details) increase considerably as the wiring cost efficient *η* rises. Both metrics peak near the region for which network performance is optimal and, as *η* is increased further, *Q*, *C* and predictive performance all decline. These phenomena are consistent across rewiring methods, suggesting trade-off between network performance, topological features, and wiring cost as a broadly applicable guiding principle for neural network architecture search.

### Development of task-specific modules

**Fig. 3.**
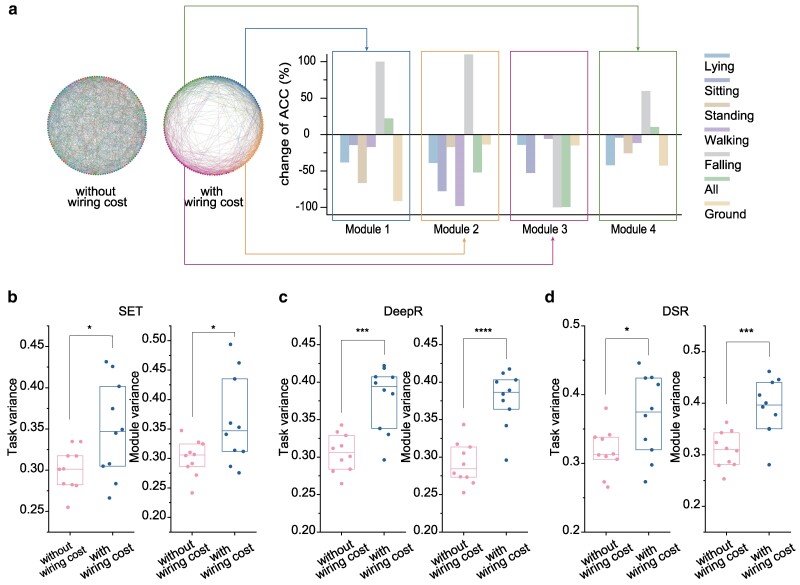
Emergence of task-related structural modules. a) Visualization of wiring diagrams of the hidden units for GRU models trained with (right) and without (left) wiring cost. The wiring diagram consists of 131 units and 1,500 connections, where the units self-organize into different modules identified by the Louvain method (49), with each unit color-coded based on its module assignment. The histogram illustrates a lesion experiment on a representative GRU, where neurons within the same module and their connections are removed to assess the change of accuracy for each subtask on the test dataset without retraining. b–d) Comparison of average task variance or average module variance between GRUs trained with and without wiring cost are detailed using three different rewiring strategies (SET, DeepR, and DSR) on the HAR dataset, which consists of seven subtask categories: Lying, Sitting, Standing, Walking, Falling, All, and Ground. Each point in the plot represents a trial, and box plots present upper quartile, median, and lower quartile values over 10 experiments. Significance levels 5, 1, 0.1, and 0.01% are indicated by one, two, three, and four asterisks, respectively, determined using the two-sample *t*-test.

The relationship between structural modules and functional specialization has a strong impact on the interpretability of learning (50–52). To explore the congruence between structural modularity and functional specialization, we now employ lesion experiments on optimized sparse GRUs. In Fig. 3a, we illustrate examples of GRUs trained on the HAR dataset with modules identified using the Louvain method (49). Under our cost-controlled framework four distinct modules emerge, but this feature is absent when models are trained based on task performance alone (see Methods for more details). Next, we consider dropping out all neurons and assessing the resulting changes in test accuracy for each of the sub-tasks. We observe striking variation in the extent to which lesioning a module affects performance in a subtask. For example, removing modules 1, 2, or 3 results in a significant drop in prediction accuracy for Ground, Walking, Falling, and All sub-tasks. In fact, the damaged model almost loses its ability to recognize these tasks. In contrast, for the Falling sub-task, removing module 1 or 2 has no adverse effect, and can even improve prediction performance.

To quantify the correlation between structural modules and sub-tasks, we introduce two metrics: task variance and module variance. Task variance measures the variation in performance across various sub-tasks when a specific module *β* is damaged (i.e. removing all neurons and their connected connections in the module) and can be evaluated as follows:


(2) $$\sigma_{\mathrm{task}}^{2}(\beta )=\langle [\text{acc}(i,\beta )-\langle ,\text{acc}(i^{\prime },\beta )\rangle_{i^{\prime }}{]}^{2}\rangle_{i}$$

where acc(i,β) represents the relative change in performance for sub-task *i* after module *β* is removed, and $\langle \cdot \rangle_{x}$ represents the average over variable *x*. A large value of $\sigma_{\mathrm{task}}^{2}(\beta )$ indicates that module *β* is highly specialized for one of the sub-tasks, while a low value suggests that its has a more equal role in different sub-tasks. In contrast, module variance measures the difference in performance change of the same sub-task after different modules have been damaged. The module variance for sub-task *i* is:


(3) $$\sigma_{\mathrm{module}}^{2}(i)=\langle [\text{acc}(i,\beta )-\langle \text{acc}(i,\beta^{\prime })\rangle_{\beta^{\prime }}{]}^{2}\rangle_{\beta }.$$

A high value of $\sigma_{\mathrm{module}}^{2}(i)$ suggests that sub-task *i* depends on one of the modules, while a small value signifies that the representation of sub-task *i* is driven by the cooperation of all these modules.

We compare the average task variance and average module variance for sparse GRUs trained with and without wiring cost. The average task variance $\langle \sigma_{\mathrm{task}}(\beta )\rangle_{\beta }$ is calculated across all modules, while the average module variance $\langle \sigma_{\mathrm{module}}(i)\rangle_{i}$ is calculated across all sub-tasks. Figure 3b–d reveals a pronounced elevation in both average task variance and average module variance across the rewiring algorithms incorporating wiring cost constraints: in all cases, increases in average variance are significant at the 5% level and, in half of cases, significant at the 0.1% level. These findings establish that modular structure which arises as a by-product of networks minimizing wiring cost is relevant to the tasks being performed and aids interpretation of ANNs.

### Matching biological network geometry while achieving high task performance

So far, we have been instantiating neurons within classical geometric shapes. However, spatially embedded networks, such as brain networks, typically arise in more complex spaces. In this section, we aim to use the wiring cost-controlled framework to replicate geometric features observed in intricate biological networks.

**Fig. 4.**
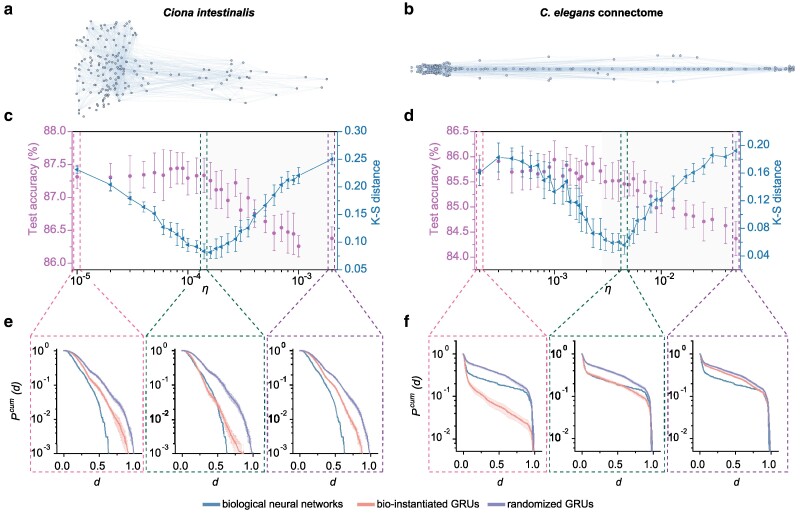
Imitating nature by minimizing wiring cost. The bio-instantiated GRU, which has a single hidden layer inspired by either a) the central nervous system (CNS) of *Ciona intestinalis* or b) the connectome of *C. elegans*, are trained on the HAR dataset using DeepR. c, d) Variation with wiring cost coefficient *η* of model test accuracy (left *y* axes, rose red circles) and K–S distance (right *y* axes, blue triangles) between distribution of interneuronal connection distance of bio-instantiated GRUs and distribution of real biological organisms. Lower K–S distance values indicate a higher similarity between the models. In the shaded areas, network performance decreases, and K–S distance tends to increase, with wiring cost coefficient *η*. e, f) Comparison of the cumulative distribution of interneuronal distances *d* between optimized bio-instantiated GRUs (orange line) and the neuronal network of *Ciona intestinalis*’ CNS or *C. elegans* connectome (blue line). For comparison, we also show the corresponding randomized GRUs (purple line), in which all connections in the optimized bio-instantiated GRUs remain unchanged, but neuron locations are randomly rearranged. Interneuronal distances *d* are normalized by the longest observed separation. Plots show the average over 10 independent runs, error bars represent standard deviation, and shaded intervals represent 95% CI.

To achieve this, we incorporate anatomically detailed neural maps of the central nervous system of *Ciona intestinalis* (53) (consisting of 177 neurons and 1,920 connections, see Fig. 4a) and the *C. elegans* connectome (54) (consisting of 279 neurons and 2,194 connections, see Fig. 4b). These neural maps provide detailed biological information for constructing bio-instantiated GRU, including the spatial coordinates and layout of neurons. Specifically, our bio-instantiated GRUs are designed with a single hidden layer with the same number of neurons and links as the real organism’s neural map, and with neuron locations identical to those in the corresponding organism (see Methods for more details). In Fig. 4c and d, we consider varying the wiring cost coefficient *η* as we train bio-instantiated GRUs on the HAR dataset using DeepR rewiring algorithm within our wiring cost-controlled framework, and employ the Kolmogorov–Smirnov (K–S) distance (55) metric to quantify the difference in distributions of interneuronal connections between optimized bio-instantiated GRUs and real biological neural networks. We visualize these differences in Fig. 4e and f, where we juxtapose the cumulative distributions of interneuronal connection distances among the observed biological network, trained bio-instantiated GRUs and, as a baseline, randomized GRUs in which all connections in the optimized bio-instantiated GRUs remain unchanged, but the neuron locations are randomly rearranged. For small values of *η*, the predictive performance of bio-instantiated GRUs can initially improve slightly as *η* increases. However, beyond a certain range, network performance gradually declines as *η* grows. K–S distance shows a distinct pattern of variation with *η*. For small values of *η*, the K–S distance is high, and optimized bio-instantiated GRUs can easily be distinguished from biological neural networks on the basis of interneuronal separation (Fig. 4e, left and f, left). However, as the value of *η* increases, the disparity between bio-instantiated GRUs and real biological neural networks diminishes, while the predictive performance of the bio-instantiated GRUs remains stable within this range. We see an optimal interval of wiring cost in which bio-instantiated GRUs exhibit both high network performance and a close resemblance to the wiring patterns observed in real biological networks (Fig. 4e, middle and f, middle). Beyond this range, the disparity in wiring between the bio-instantiated GRUs and biological networks gradually increases with higher values of *η* and, simultaneously, network performance declines (Fig. 4e, right and f, right). These findings suggest that the wiring patterns observed in biological neural networks, which have undergone cost optimization through evolution, offer a promising avenue for investigating optimal architectures in ANNs.

## Discussion

In this article, we explored wiring economics within artificial neural networks (ANNs), drawing inspiration from the intricate organization of brain networks. Through the utilization of a wiring cost-controlled training framework that concurrently optimizes wiring efficiency and task performance for sparse ANNs, we have uncovered the pivotal role of wiring cost in the architecture and refinement of ANNs. Our investigation has demonstrated the positive impact of wiring cost for enhancing network performance, elevating task learning capabilities, and facilitating the emergence of modular network topologies for ANNs whose neurons have arbitrary but fixed spatial locations. In addition to sparse ANNs with recurrent architectures, the wiring cost-controlled training strategy also improves network performance in feed-forward neural networks (see Supplementary Information Section VI). By incorporating geometric constraints into spatially realized ANNs, we have achieved a remarkable replication of the connection distance distribution observed in maps of real organisms. This replication is pronounced when ANNs attain high task performance levels, offering new insights into the fundamental principles of biological organization.

Our cost-control framework has parallels with $L_{1}$ regularization (46), while a variant (see Supplementary Information Section V) has links to $L_{2}$ regularization (47). Therefore, some of the improvements provided by cost control are likely due to the mechanisms which support $L_{1}$ and $L_{2}$ regularization. For example, $L_{1}$ regularization, by driving many weights to zero, helps the model focus on important features and discourages overfitting, while $L_{2}$ regularization, by discouraging large weights, encourages robustness and stability (56). However, as mentioned above, wiring cost control can provide benefits even relative to $L_{1}$ and $L_{2}$ regularization (see also Supplementary Information, Sections IV and V). We posit these additional advantages arise because the geometric structure entailed by wiring cost control reduces the effective search space, making optimization easier. Another possible source of improvements could be that weighting by distance eliminates symmetries which, in $L_{1}$ and $L_{2}$ regularization, could make certain optimization directions redundant with respect to one another and thus engender inefficiencies in search.

Moving forward, there are several promising avenues for future research. Advances in experimental technology are making available more time series from collections of neurons whose connectivity is also known (57), and it would be interesting to seek wiring cost-based training principles which allow ANNs to emulate both activity and topology of these biological neuronal networks. Also, delving into the trade-offs between wiring cost and other crucial properties of network connectivity, including topological efficiency, robustness, and connector hubs, will provide a more comprehensive understanding of network architectures. Additionally, investigating the mysteries of brain network organization and leveraging these insights to inform the design of ANNs holds great potential. By incorporating principles inspired by natural networks into the design process (58), we can unlock new possibilities for developing more intelligent and interpretable models architectures and learning strategies.

## Methods

### Wiring cost

The concept of wiring cost originates from the biological nervous system and arises from factors such as the establishment and maintenance of synaptic connections between neurons, as well as the metabolic energy consumed during signal transmission along these connections. While the exact biological wiring cost remains unresolved, it can be inferred that the farther apart two neurons are, the more costly will tend to be the connection between them. Accordingly, metrics such as wiring volume provides approximate measures for evaluating the wiring cost of neural networks (Fig. 1b).

For a neural network with *n* neurons and *m* links, the wiring cost $C_{\mathrm{wiring}}$ is calculated as the sum of the Euclidean distances between neurons, multiplied by the strength of their connection (31)


(4) $$C_{\mathrm{wiring}}=\sum_{i=1}^{n}\sum_{\;j=1}^{n}A_{ij}D_{ij}\cdot |w_{ij}|,$$

or a normalized alternative


(5) $$C_{\mathrm{wiring}}=\frac{\frac{1}{m}\sum_{ij}A_{ij}\cdot |w_{ij}|D_{ij}}{\sqrt{\frac{1}{m}\sum_{ij}\cdot |w_{ij}{|}^{2}}\sqrt{\frac{1}{n(n-1)}\sum_{ij}D_{ij}^{2}}},$$

where $A_{ij}$ represents an element in the adjacency matrix *A* of the network, indicating whether there is a connection from neuron *i* to *j*, $D_{ij}$ corresponds to the Euclidean distance between neuron *i* and *j*, and $w_{ij}$ is the weight of the connection from neuron *i* to *j*. Weights $w_{ij}$ can be positive or negative, representing excitatory or inhibitory connections, respectively.

### Recurrent network models

#### Recurrent neural networks

Recurrent neural networks (RNNs) have cyclic connections that enable hidden units to use previously calculated outputs as inputs for subsequent time steps. Given an input sequence $x=[x_{1},x_{2},\ldots ,x_{T}]$ and its corresponding output sequence $O=[o_{1},o_{2},\ldots ,o_{T}]$, the hidden state at time step *t* is defined as


(6) $$h_{t}=\text{ReLU}(Ux_{t}+Wh_{t-1}+b),$$

where *U* and *W* represent the input and recurrent connection matrices, respectively, while *b* is the bias, and ReLU (59) is the activation function. The input features $x_{t}$ are fed to each recurrent unit through a linear function $Ux_{t}$. The output of RNNs at time step *t* is determined by


(7) $${\hat{o}}_{t}=\sigma (Vh_{t}+c),$$

where $\sigma (x)=1/(1+e^{-x})$ is the logistic function, *V* is the output connection matrix, and *c* is the bias. To sparsify the hidden layer, we carefully prune the connections (weights) by employing a binary mask with the same size and shape as the weight tensor *U* for the recurrent layer, effectively preventing these connections from partaking in the forward execution of the computational graph.

#### Neural ordinary differential equation

The ordinary differential equation (ODE) network is a specific class of deep neural network models that captures the continuous dynamics of hidden units by parameterizing them with an ordinary differential equation informed by the neural network, instead of specifying a discrete sequence of hidden layers. The equation governing the hidden state $h_{t}$ is defined as


(8) $$\frac{dh_{t}}{dt}=f(h_{t},x_{t},t,\theta ),$$

where *f* is a neural network parameterized by *θ*, and $x_{t}$ is the input feature at time *t*. In our experiments, we employed the fourth-order Runge–Kutta method to numerically solve the differential equations underlying the neural ODEs.

#### Continuous-time recurrent neural networks

CT-RNNs are deep learning models that approximate dynamical time-variant systems by incorporating the continuous evolution of hidden states between observation times. Instead of explicitly defining the update function for the hidden state $h_{t}$, CT-RNNs follow an ordinary differential equation specified as


(9) $$\frac{dh_{t}}{dt}=-\frac{h_{t}}{\tau }+f(h_{t},x_{t},t,\theta ),$$

in which *f* is the neural network parameterized by *θ*, and $x_{t}$ is the input at time *t*. The term $-\frac{h_{t}}{\tau }$ facilitates the autonomous system in reaching an equilibrium state and *τ* is a time constant. CT-RNNs can be trained using backpropagation through time (BPTT) along with ODE solvers.

#### Gated recurrent unit

The fundamental architecture of the GRU includes two main gates, i.e. the reset gate $r_{t}$ and the update gate $s_{t}$. The reset gate controls the proportion of the previous hidden state $h_{t-1}$ that contributes to the candidate state ${\tilde{h}}_{t}$. The update gate determines the balance between retaining previous information and incorporating new input, controlling both the preservation of the previous state and the integration of the candidate state. By combining the reset and update gates, the GRU can dynamically update its hidden state, adapting to the current input $x_{t}$ and the previous hidden state. The equations governing the GRU are as follows:


(10) $$\begin{array}{cc} & r_{t}=\sigma \left(W^{R}x_{t}+U^{R}h_{t-1}+b^{R}\right)\\ & \;\;\;\;\;{\tilde{h}}_{t}=\text{tanh}\left(W^{Q}x_{t}+U^{Q}\left(r_{t}⊙h_{t-1}\right)+b^{Q}\right)\\ & \;\;s_{t}=\sigma \left(W^{S}x_{t}+U^{S}h_{t-1}+b^{S}\right)\\ & h_{t}=\left(1-s_{t}\right)⊙h_{t-1}+s_{t}⊙{\tilde{h}}_{t}, \end{array}$$

where *σ* represents the sigmoid function defined as $\frac{1}{1+e^{\left(-x\right)}}$. The matrices $W^{x}$, $U^{x}$, and vectors $b^{x}$, where x∈{R,Q,S}, serve as the model parameters. The term ⊙ denotes the Hadamard product defined by $\left(a_{ij})⊙(b_{ij})=(a_{ij}b_{ij}\right)$, and the initial hidden state is $h_{0}=0$.

### Dynamic sparse rewiring strategies

#### Sparse evolutionary training

SET (10) employs magnitude-based pruning and regrowth techniques to reorganize the connections between neurons in a sparse network. At the end of each training epoch, a fraction *ζ* of the positive and negative connections with the smallest magnitudes is pruned. Subsequently, new connections equal in number to the pruned connections, are randomly added and initialized to 0. During the evolution of the network structure, the total number of connections remains fixed. The parameter *ζ* used in our experiments is presented in Supplementary Information Table S1.

#### Deep rewiring

DeepR (39) is a sparse network rewiring algorithm with a Bayesian theoretical foundation. It formulates the rewiring process as a stochastic sampling of network configurations from the posterior distribution and ensures strict control over the total number of connections within the sparse network throughout the training process. During the rewiring phase, DeepR assigns a predefined sign $s_{i}\in \{-1,1\}$ to each connection *i*. The weight of each active connection *i* (i.e. a connection *i* with nonzero weight $w_{i}$) is updated according to the strategy


(11) $$w_{i}^{\prime }=w_{i}-\alpha \frac{\partial L\left(\;f(X,\theta );Y\right)}{\partial w_{i}}-\alpha \xi +\sqrt{2\eta T}\nu_{i},$$

where *α* represents the learning rate, L(f(X,θ);Y) refers to the loss function involving the input *X*, target output *Y*, and model parameters θ are computed using backpropagation. The term −αξ functions as an $L_{1}$ regularization term. DeepR combines gradient descent with a random walk by introducing a noise term $\sqrt{2\alpha T}\nu_{i}$ that enables random fluctuations within the parameter space. In this equation, $\nu_{i}$ denotes a standard Gaussian random variable, and *T* controls the strength of the noise. If the sign of $w_{i}^{\prime }$ differs from $s_{i}$, connection *i* will be deactivated, and its updated weight is set to $w_{i}^{\prime }=0$. Once a connection is deactivated, a new connection $i^{\prime }$ is randomly chosen from the inactive connections, activated, and initialized with $w_{i^{\prime }}=s_{i^{\prime }}\epsilon$, where $\epsilon =1\times 10^{-12}$. Hyperparameter settings for DeepR are presented in Supplementary Information Table S1.

#### Dynamic sparse reparameterization

Dynamic sparse reparameterization (DSR) (40) utilizes dynamic parameter reassignment to optimize sparse network structure. In the pruning phase, weights are compared to a global threshold *H*. If the percentage of pruned parameters exceeds (1+δ)K, where *K* represents the target number of parameters to be pruned with a fractional tolerance of *δ*, the pruning threshold *H* is halved. Conversely, if the percentage of pruned parameters falls below (1−δ)K, the pruning threshold is doubled. This adaptive adjustment of *H* ensures that approximately *K* weights are globally pruned per iteration.

During the growth phase, DSR redistributes the number of parameters across layers using a specific strategy, in which layer *l* is assigned growth parameters that satisfy


(12) $$G_{l}^{\left(t\right)}=\frac{R_{l}^{\left(t\right)}}{\sum_{l}R_{l}^{\left(t\right)}}\sum_{l}K_{l}^{\left(t\right)},$$

where $K_{l}^{\left(t\right)}$ and $R_{l}^{\left(t\right)}$ are the count of pruned and surviving parameters, respectively. This strategy allocates more free parameters to layers with more surviving connections while maintaining global sparsity by balancing the number of parameters being pruned and grown. The hyperparameters of DSR are set as follows: H=0.001, δ=0.1, and *K* takes different values based on the models and datasets. For recurrent network models such as RNN, CT-RNN, Neural ODE, and GRU, *K* is set to 100 for the HGS and MNIST datasets, while the HAR dataset uses K=250.

### Network metrics

#### Modularity

Modularity is a crucial metric used to evaluate the effectiveness of community division in networks. It quantifies the difference in the density of connections within and between communities. A higher modularity signifies a greater number of connections within a community compared with connections between communities. The modularity *Q* of a network with *c* communities is defined as (60)


(13) $$Q=\frac{1}{2m}\sum_{ij}\left(A_{ij}-\frac{k_{i}k_{j}}{2m}\right)\delta \left(c_{i},c_{j}\right),$$

where $A_{ij}$ represents the number of edges between nodes *i* and *j*, which is typically either 0 or 1. The variables $k_{i}$ and $k_{j}$ denote the degrees of the respective nodes, and $m=\frac{1}{2}\sum_{i}k_{i}$ is the total number of edges in the network. The communities to which nodes *i* and *j* belong are denoted by $c_{i}$ and $c_{j}$, respectively. The Kronecker delta function, denoted by *δ*, is defined as δ(x,y)=1 if x=y and 0 otherwise.

#### Clustering coefficient

The clustering coefficient reflects the extent of clustering among vertices in a network. In the case of an undirected network, the local clustering coefficient of a node *i* quantifies the ratio of directly connected node pairs among the neighbor nodes of node *i* to the total number of neighbor node pairs of node *i*. This can be calculated using the adjacency matrix *A* as


(14) $$C_{i}=\frac{1}{k_{i}(k_{i}-1)}\sum_{\;j,k}A_{ij}A_{\;jk}A_{ki},$$

where $k_{i}$ represents the number of links (i.e. degree) connected to node *i*. When $k_{i}$ is either zero or one, we impose $C_{i}=0$. The overall level of clustering in the network is determined by calculating the average of the local clustering coefficients of all *n* nodes.

### Module identification by Louvain method

We use the Louvain algorithm to identify the modular organization within the optimized networks. Louvain (49), a module detection algorithm proposed for large networks, discerns the network’s modular structure by maximizing modularity. This method operates as a greedy optimization approach with a time complexity of O(n⋅logn), where *n* represents the number of nodes in the network, capable of uncovering hierarchical modular arrangements. In our lesion experiments, we use the Louvain method to partition the structural modules of models trained under wiring cost constraints and without them. The model trained with wiring cost exhibits easily discernible modules, and modularity is maximized when the number of modules is modest. Conversely, the model trained without considering wiring cost displays indistinct modular structures featuring a larger number of modules at optimum modularity.

### Maps of biological organisms

#### *Caenorhabditis elegans* connectome

The wiring diagram of the *C. elegans* nematode (54) displays a hierarchical network topology. External signals are received through sensory neurons, which subsequently transmit these signals to interneurons and command neurons for decision making. Finally, decisions are relayed to motor neurons which regulate muscle movement. The wiring diagram of *C. elegans* exhibits a high level of sparsity, with 299 neurons interconnected through 2,279 neuron connection pairs. Each neuron connection pair is connected by one or more synapses, resulting in a total of approximately 6,465 connections.

To construct the bio-instantiated GRU, we draw on the largest connected subgraph of the *C. elegans* connectome, which consists of 279 neurons interconnected by 2,194 neuronal connection pairs. This subgraph includes 86 sensory neurons and 118 motor neurons, each of which has 3D spatial coordinates. The input features are fed into the bio-instantiated GRU through the units corresponding to the sensor neurons, while the output of the bio-instantiated GRU is transmitted to the output neurons via the hidden units associated with the motor neurons.

#### *Ciona intestinalis* connectome

*Ciona intestinalis* is among the few organisms with a fully resolved connectivity map. Through serial-section electron microscopy, the synaptic connectomes of 177 central nervous system (CNS) neurons in larvae of *Ciona intestinalis* have been experimentally recorded (53). The chemical synaptic network of CNS neurons consists of 177 nodes and 1,920 node connection pairs, with a clustering coefficient of 0.335 and a mean network distance of 2.541. Based on this synaptic network, the bio-instantiated GRU is constructed with a sparse hidden layer consisting of 177 nodes and 1,920 connections. Input features are fed to each hidden unit, and the output of the bio-instantiated GRU is transmitted to the output neuron through a learnable linear layer.

### Description of datasets

#### Sequential MNIST

MNIST is a classic dataset composed of 28×28 pixel grayscale images of handwritten digits. In our experiments, we convert each sample into a 28D time series with length 28, and scale all input features to the range [0,1]. MNIST has a training set of 60,000 examples and a test set of 10,000 examples. In our experiments, we partitioned 20% of the training dataset (i.e. 12,000 examples) into a validation set.

#### Hand gesture segmentation

The HGS dataset consists of features extracted from seven videos depicting people gesticulating. Each sample comprises 32 input features and a corresponding output phase which can be either rest position, preparation, stroke, hold, or retraction. The dataset is divided into a training set, a validation set and a test set using the ratios 0.75:0.15:0.1. Furthermore, we divide the sequences within each of these sets into overlapping subsequences, where each subsequence consists of exactly 32 time steps. During training, we evaluate the network’s performance on the validation set after every epoch and save the weight configuration that achieves the best validation metric. Once training is complete, we restore the previously saved optimal weights and assess the network’s performance on the test set.

#### Human activity recognition

The human activity (HAR) dataset consists of recordings from 25 human participants engaging in various physical activities such as walking, lying down, and standing up from sitting. Each participant was equipped with four different sensors, and each sensor captured data at a sampling period of 2D11 ms. Similarly to Hasani et al. (61), we represent the input time series as a 7D feature vector, where the first four items represent the sensor ID and the last three items represent the sensor value. The 11 activity items are classified into 7 distinct categories. The dataset is divided into a training set, a validation set and a test set using the ratios 0.75:0.15:0.1. The validation and test metrics are computed after every training epoch. The network’s performance is evaluated by considering the classification accuracy on the test set at the epoch with the highest validation accuracy during training.

## Supplementary Material

pgae580_Supplementary_Data

## Data Availability

All datasets used in this article are freely available online and can be accessed as follows: https://archive.ics.uci.edu/dataset/302/gesture+phase+segmentation for Hand gesture segmentation; http://yann.lecun.com/exdb/mnist/ for MNIST; https://archive.ics.uci.edu/ml/machine-learning-databases/00196/ConfLongDemo_JSI.txt for Human activity recognition; https://github.com/gyyang/multitask for the naturalistic cognitive task; http://www.cs.toronto.edu/~kriz/cifar.html for CIFAR-10; https://github.com/zalandoresearch/fashion-mnist for Fashion-MNIST; https://github.com/3BIM20162017/CElegansTP for the connectome of *C. elegans*. The code to reproduce all experiments is available online at https://github.com/XinjieZhang/wiring-cost-of-ANNs and on Zenodo at https://doi.org/10.5281/zenodo.10911579.
