# Supplementary material for: Brain-inspired wiring economics for artificial neural networks
Source: PNAS Nexus. 2025 Jan 7;4(1):pgae580. doi: 10.1093/pnasnexus/pgae580 (PMC11736432; doi:10.1093/pnasnexus/pgae580)
Supplement: pgae580_Supplementary_Data [file pgae580_supplementary_data.pdf]

Brain-inspired wiring economics for artificial neural networks

Contents

I Spatial implementation of artificial neural networks 2

II Varying network sparsity 3

III Naturalistic tasks 5

IV Comparison of wiring cost control and regularization 7

V Statistical significance 9

VI Compatibility with feed-forward neural networks 12

I Spatial implementation of artificial neural networks

We spatially embed artificial neural networks by assigning geometric coordinates to each neuron. In recurrent network architectures, all recurrent units are positioned randomly and uniformly along the circumference of a unit circle (Fig. S1 A). For feed-forward neural network architectures, such as multi-layer perceptrons (MLPs), we spread the neurons of hidden layers in the $x - y$-plane such that all in layer k possess a y-coordinate of k, are evenly spaced with unit interval in the x-direction, and are distributed symmetrically about the y-axis (Fig. S1 B).

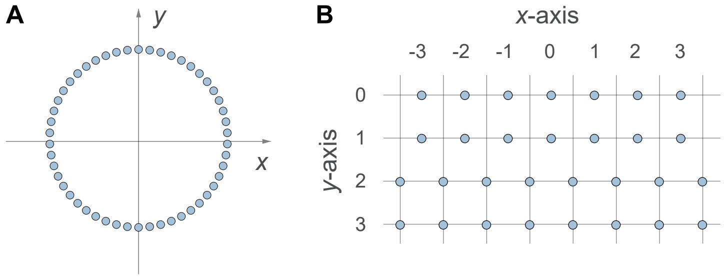

Figure S1: (A) The spatial arrangement of recurrent units within the recurrent neural network architecture in a two-dimensional space. (B) The spatial distribution of hidden layer units within the feed-forward neural network architecture in a two-dimensional space.

II Varying network sparsity

To explore the dependence of our wiring cost controlled training framework on network sparsity, in Fig. S2 we varied the sparsity level of the sparse GRU from 0.1 to 0.9 and compared the performance of these models trained on the HAR dataset without and with wiring cost. Wiring cost control improves performance across a wide range of sparsities: specifically, for 7 of 9 sparsities when using SET or DeepR (Fig. S2 A,B) and for 6 of 9 sparsities when using DSR (Fig. S2 C).

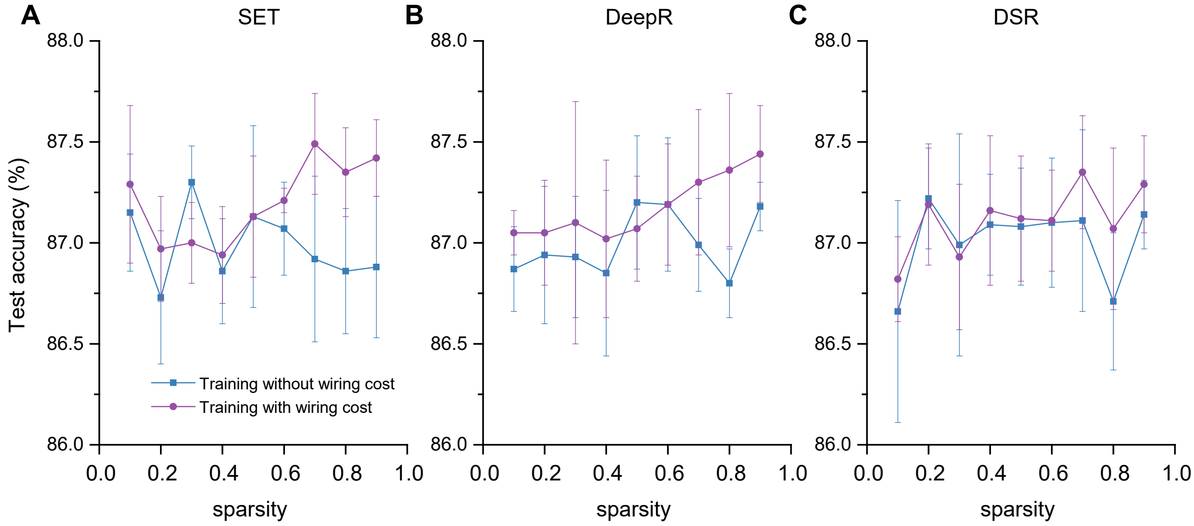

Figure S2: Performance comparison between sparse GRUs trained without (blue) and with (purple) wiring cost control at different sparsity levels. We conducted experiments on the HAR dataset using SET (A), DeepR (B), and DSR (C) algorithms. Plots show the average over 5 independent runs, and error bars represent standard deviation. Other hyperparameters used in these experiments are listed in Table S1.

Table S1: Hyperparameters for experiments involving datasets considered in the main text: HGS, MNIST and HAR.

		RNN	CT-RNN	Neural ODE	GRU
HGS		Hyperparameters for training			
	Epochs	500	500	300	300
	Batch size	32	32	32	32
	Initial learning rate	0.01	0.01	0.01	0.005
	Wiring cost coefficient<br>L_i regularization coefficient	1×10^{-4}	2×10^{-4}	4×10^{-4}	1×10^{-4}
		Hyperparameters for Sparse Evolutionary Training (SET)			
	ζ	0.1	0.2	0.2	0.1
		Hyperparameters for Deep Rewiring (DeepR)			
	α	0.01	0.01	0.01	0.005
	ξ	10^{-4}	10^{-4}	10^{-4}	10^{-4}
	T	$\frac{\alpha}{2} \times 10^{-2}$	$\frac{\alpha}{2} \times 10^{-2}$	$\frac{\alpha}{2} \times 10^{-2}$	$\frac{\alpha}{2} \times 10^{-2}$
MNIST		Hyperparameters for training			
	Epochs	200	200	100	100
	Batch size	128	128	128	128
	Initial learning rate	0.005	0.005	0.01	0.01
	Wiring cost coefficient<br>L_i regularization coefficient	4×10^{-4}	4×10^{-4}	2×10^{-4}	5×10^{-5}
		Hyperparameters for Sparse Evolutionary Training (SET)			
	ζ	0.1	0.1	0.1	0.1
		Hyperparameters for Deep Rewiring (DeepR)			
	α	0.005	0.005	0.01	0.01
	ξ	10^{-5}	10^{-5}	10^{-5}	10^{-4}
	T	$\frac{\alpha}{2} \times 10^{-4}$	$\frac{\alpha}{2} \times 10^{-4}$	$\frac{\alpha}{2} \times 10^{-4}$	$\frac{\alpha}{2} \times 10^{-2}$
HAR		Hyperparameters for training			
	Epochs	200	200	200	200
	Batch size	64	64	64	64
	Initial learning rate	0.01	0.01	0.01	0.01
	Wiring cost coefficient<br>L_i regularization coefficient	4×10^{-4}	2×10^{-4}	2×10^{-5}	1×10^{-4}
		Hyperparameters for Sparse Evolutionary Training (SET)			
	ζ	0.2	0.2	0.2	0.1
		Hyperparameters for Deep Rewiring (DeepR)			
	α	0.01	0.01	0.01	0.01
	ξ	10^{-4}	10^{-4}	10^{-4}	10^{-4}
	T	$\frac{\alpha}{2} \times 10^{-2}$	$\frac{\alpha}{2} \times 10^{-2}$	$\frac{\alpha}{2} \times 10^{-2}$	$\frac{\alpha}{2} \times 10^{-2}$

ζ is the fraction of connections to be pruned, the term $\alpha\xi$ controls the strength of regularization, T controls the strength of noise and, in L_i regularization, $i = 1, 2$.

III Naturalistic tasks

In this section, we illustrate the efficacy of the wiring cost controlled training framework in performing naturalistic cognitive tasks, namely, two collections of decision-making (DM) subtasks: the DM Five task and the Mante task [S1],[S2]. The Mante task consists of two perceptual subtasks, Ctx DM1 and Ctx DM2, which are inspired by context-dependent DM tasks performed by macaque monkeys [S1]. The DM Five task augments the subtasks of the Mante task with three additional subtasks, making a total of five perceptual subtasks: DM1, DM2, Ctx DM1, Ctx DM2 and MultSen DM. Each subtask considers 70 features from three types of noisy inputs and a 33-dimensional response output phase that represents the direction on a one-dimensional ring. For either the Mante or DM Five task, we generate training and testing datasets using the method described in Ref. [S2], which provides more details and the common setup of these naturalistic cognitive tasks.

To explore the relationship between wiring cost, network performance, and topology of sparse recurrent neural networks (RNNs) trained to perform naturalistic tasks, we consider RNNs with a single hidden layer and vary the wiring cost coefficient η from 1×10^{-5} to 5×10^{-3}. As shown in Fig. S3, for smaller values of η, the test accuracy, modularity Q and average clustering coefficient C of the network all increase with η. When the value of η is too large, test accuracy begins to decrease, and Q and C also show a consistent decreasing trend.

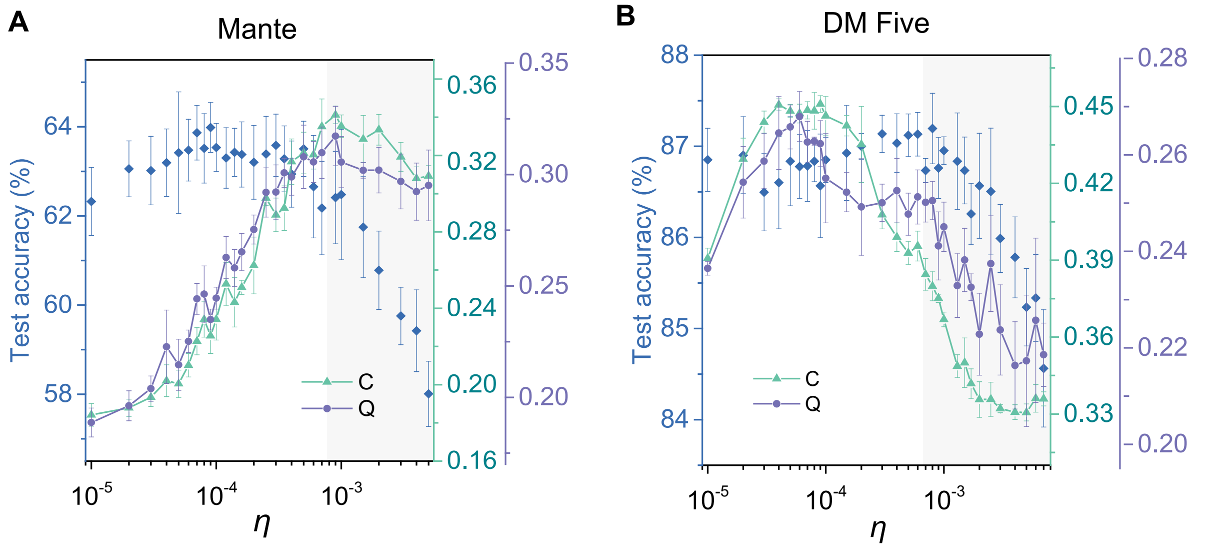

Figure S3: The test accuracy (blue diamonds), modularity (Q, purple circles) and average clustering coefficient (C, green triangles) of RNNs versus wiring cost control coefficient η for the (A) Mante and (B) DM Five naturalistic tasks. Each plot is the average of 5 independent runs, and the error bars represent standard deviation. In the shaded areas, test accuracy, Q, and C show a consistent decreasing trend. The dynamic sparse rewiring strategy SET is used. Other hyperparameters used in these experiments are listed in Table S2.

Table S2: Hyperparameters for training RNNs on naturalistic tasks Mante and DM Five.

Experiment	Mante	DM Five
	Hyperparameters for training	
Number of hidden neurons	131	256
sparsity	0.9	0.85
Training iteration	5000	10000
Batch size	64	64
Initial learning rate	0.01	0.01
	Hyperparameters for Sparse Evolutionary Training (SET)	
ζ	0.2	0.2
	Hyperparameters for Deep Rewiring (DeepR)	
α	0.01	0.01
ξ	10^{-4}	10^{-4}
T	$\frac{\alpha}{2} \times 10^{-2}$	$\frac{\alpha}{2} \times 10^{-2}$
	Hyperparameters for Dynamic sparse reparameterization (DSR)	
H	0.001	0.001
δ	0.1	0.1
K	250	1000

ζ is the fraction of connections to be pruned, the term $\alpha\xi$ controls the strength of regularization, T controls the strength of the noise, H is the global threshold, K represents the target number of parameters to be removed with a fractional tolerance of δ.

IV Comparison of wiring cost control and regularization

Our wiring cost controlled training strategy optimizes network structure in a manner akin to regularization. In this section we show how incorporating wiring cost control improves performance even relative to ANNs trained with regularization. In machine learning, overfitting can be discouraged by adding an additional regularization term to the loss function to reduce the strength of network weights. Lasso [S3] or L_1 regularization encourages the model towards sparsity by adding the L_1 norm of weights (the sum of the absolute values of weight vectors) as a penalty term in the loss function of the model, resulting in $\mathcal{L} = L_{\text{task}} + \eta \sum_{ij} |w_{ij}|$, where L_{task} is the loss function for the task, w_{ij} is the weight of the model and η is the regularization coefficient. Ridge [S4] or L_2 regularization is achieved by adding the L_2 norm of weights (the sum of squares of weight vectors) to the loss function of the model, resulting in $\mathcal{L} = L_{\text{task}} + \eta \sum_{ij} |w_{ij}|^2$. The regularization coefficient η is designed to balance the fitting degree of training data and the complexity of the model. If η is too large then the model may neglect important features, while η which is too small can allow overfitting.

Table S3: Value of wiring cost coefficient η or L_i $(i = 1, 2)$ regularization coefficient.

Experiment	Mante	DM Five
SET	1×10^{-4}	3×10^{-4}
DeepR	1×10^{-4}	1×10^{-4}
DSR	3×10^{-4}	3×10^{-4}

Here, we incorporate the wiring cost constraint into L_i (where $i = 1, 2$) regularization, leading to $\mathcal{L} = L_{\text{task}} + \eta \sum_{ij} |w_{ij}| \cdot \|r_i - r_j\|$ for L_1 combined with wiring cost and $\mathcal{L} = L_{\text{task}} + \eta \sum_{ij} |w_{ij}|^2 \cdot \|r_i - r_j\|$ for L_2 combined with wiring cost. As shown in Fig. S4, we compare the performance under L_1 and L_2 regularization with and without a wiring cost constraint, using equal values for the L_i regularization coefficient and wiring cost coefficient, as shown in Table S1,S3. Incorporating wiring cost constraint improves the mean performance of L_1 (L_2) regularization in 19 of 24 (18 of 24) cases, which is significant at the 0.5% (5%) level based on a null binomial model with $n = 24$ trials and success probability $p = 0.5$. In fact, these levels of significance, each based on 24 pairs of mean values of performance, do not fully reflect the consistency of the improvement from incorporating wiring cost constraint into L_i regularization. More sophisticated statistical analysis reveals improvements significant at the 10^{-5} level for L_1 and the 5×10^{-4} level for L_2 (see Sec. V).

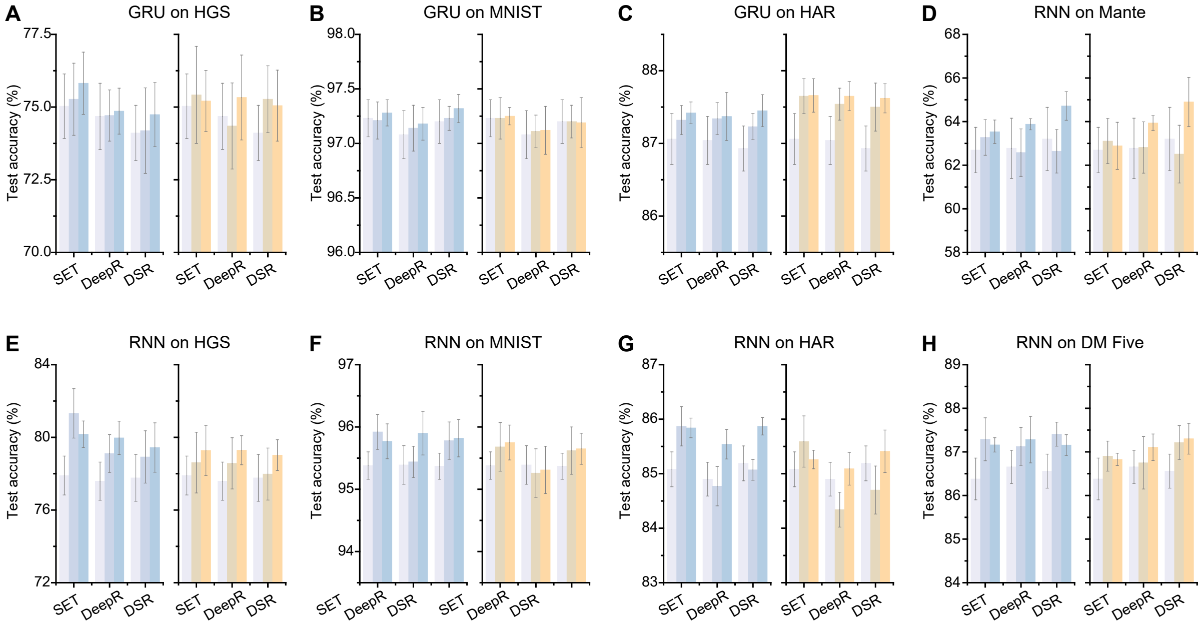

Figure S4: Impact of L_i $(i = 1, 2)$ regularization and wiring cost control of performance of GRU (A-C) and RNN (D-G) models. We trained each model on the HGS, MNIST, and HAR datasets using three dynamic sparse rewiring strategies: SET, DeepR and DSR. Hyperparameters used in these experiments are listed in Table S1,S2,S3. The model trained without regularization or cost control is used as the baseline. Each point is the average of either (A-C,E-G) 10 or (D,H) 5 independent runs, and error bars represent the standard deviation.

V Statistical significance

In this section we provide additional statistical evidence that incorporating wiring cost constraint into training enhances performance. We do so by testing, for norms $i = 1, 2$, the null hypothesis

H_0^i: For a given combination of dataset, model and training strategy, the test accuracy under training strategies L_i and L_i with wiring cost constraint (in this section abbreviated to L_i+wiring) arise independently under the same distribution.

against the alternative hypothesis

H_1^i: For a given combination of dataset, model and training strategy, the expected test accuracy under training strategy L_i+wiring is higher than under training strategy L_i.

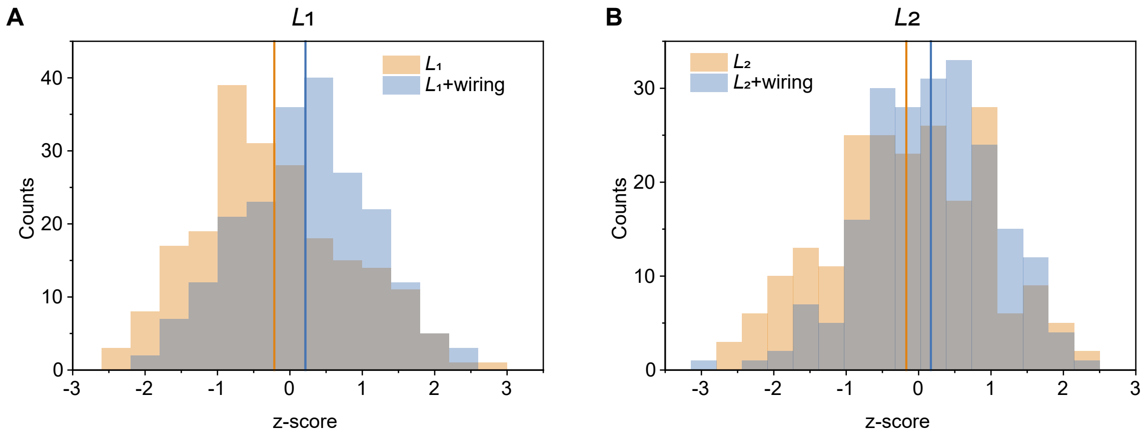

Figure S5: Histogram of z-scores for L_i (orange) and L_i+wiring (blue) where (A) $i = 1$ and (B) $i = 2$. The vertical lines show the mean z-scores under L_i (orange) and L_i+wiring (blue).

We use as discriminating statistic a version of the t-statistic calculated from pooled z-scores of test accuracy. More precisely, let condition c denote a combination of dataset, model and training strategy, and let $a_{i,c,t}^j$ denote the test accuracy in trial number j of the n_c trials under norm i, condition c, and training strategy t. For each norm i and condition c, we convert each test accuracy $a_{i,c,t}^j$ to a z-score $z_{i,c,t}^j$ calculated relative to the set $\bigcup_{j=1}^{n_c} \bigcup_{t \in \{L_i, L_i+\text{wiring}\}} \left\{ a_{i,c,t}^j \right\}$, i.e., while calculating means and variances by combining all trials and training strategies, but not combining different conditions. We then calculate a t-statistic by pooling these z-scores across distinct conditions c, i.e., based on the sets $\bigcup_{c,j} \left\{ z_{i,c,L_i}^j \right\}$ and $\bigcup_{c,j} \left\{ z_{i,c,L_i+\text{wiring}}^j \right\}$. The pooled z-scores are shown in Fig. S5, while Table S4 lists t-statistics and corresponding one-sided t-test p-values for both the L_1 and L_2 norms.

Table S4: Discriminating statistic and p-values.

Norm	t-statistic	p-value	
		t-test	surrogate testing
L_1	4.51	4.2×10^{-6}	$9. \times 10^{-6}$
L_2	3.52	2.4×10^{-4}	3.3×10^{-4}

The p-value associated with the t-test lacks theoretical justification because it arises under

the null hypothesis that all instances of z^j_{i,c,L_i} and $z^j_{i,c,L_i+\text{wiring}}$ are drawn independently from the same Gaussian distribution. However, under our null hypothesis H^i_0, although $a^j_{i,c,t}$ are independent, this does not imply that $z^j_{i,c,t}$ are. Also, although we have a large total number of samples, we cannot directly use the central limit to justify a Gaussian distribution of mean z-scores and thus calculate p-values for two reasons: (1) as we just stated, values of $z^j_{i,c,t}$ are not independent; and (2) our null hypothesis H^i_0 does not imply that values of $z^j_{i,c,t}$ are drawn from the same distribution across different c. To obtain rigorous p-values we therefore turn to the method of surrogates.

To generate from our original observation $(a^j_{i,c,t})$ a surrogate representing H^i_0, we proceed as follows. For each condition c, we create the sequence

$$\tilde{a}^1_{i,c,L_i}, \tilde{a}^2_{i,c,L_i}, \ldots, \tilde{a}^{n_c}_{i,c,L_i}, \tilde{a}^1_{i,c,L_i+\text{wiring}}, \tilde{a}^2_{i,c,L_i+\text{wiring}}, \ldots, \tilde{a}^{n_c}_{i,c,L_i+\text{wiring}}$$

by randomly shuffling the sequence of observed test accuracies across all trials j of both training methods t and a single condition c,

$$a^1_{i,c,L_i}, a^2_{i,c,L_i}, \ldots, a^{n_c}_{i,c,L_i}, a^1_{i,c,L_i+\text{wiring}}, a^2_{i,c,L_i+\text{wiring}}, \ldots, a^{n_c}_{i,c,L_i+\text{wiring}}.$$

Our surrogate $(\tilde{a}^j_{i,c,t})$ is then the collection of $\tilde{a}^j_{i,c,t}$ across each combination of trial j and condition c.

Because the surrogate method involves choosing new sequences $(\tilde{a}^j_{i,c,t})$ uniformly at random from a set of sequences, we can confirm the method represents H^i_0 regardless of unknown parameters by verifying the three conditions (C1)-(C3) for a constrained surrogate[S5]. Condition (C1) is that the surrogate method can return the original sequence $(a^j_{i,c,t})$, which is indeed the case. Condition (C2) is that the set of surrogates reachable from a generated surrogate is the same as the set reachable from the original sequence, which is also true. Condition (C3) is that the surrogate preserves likelihood under the null hypothesis. To check this, we let $f_{i,c}$ denote the (unknown) probability distribution of accuracy under norm i and condition c. Under H^i_0, the probability $\tilde{\mathcal{L}}$ that the surrogate $(\tilde{a}^j_{i,c,t})$ would arise as an observed collection of performances is then

$$\tilde{\mathcal{L}} = \prod_c \prod_{t\in\{L_i, L_i+\text{wiring}\}} \prod_{j=1}^{n_c} f_{i,c}(\tilde{a}^j_{i,c,t}). \tag{S1}$$

Because the surrogate is just a reshuffling of the original observation $(a^j_{i,c,t})$ which fixes i, c for each combination of c, we have

$$\prod_{t\in\{L_i, L_i+\text{wiring}\}} \prod_{j=1}^{n_c} f_{i,c}(\tilde{a}^j_{i,c,t}) = \prod_{t\in\{L_i, L_i+\text{wiring}\}} \prod_{j=1}^{n_c} f_{i,c}(a^j_{i,c,t}).$$

Therefore, by Eq. (S1), the probability $\tilde{\mathcal{L}}$ can be written

$$\tilde{\mathcal{L}} = \prod_c \prod_{t\in\{L_i, L_i+\text{wiring}\}} \prod_{j=1}^{n_c} f_{i,c}(a^j_{i,c,t}),$$

and the right-hand-side is the probability of the original observation $(a^j_{i,c,t})$.

We have demonstrated that the surrogate we outlined is a constrained surrogate for H^i_0, in the sense of Ref.[S5]. Therefore, we can employ any test statistic to perform hypothesis tests which are exact, i.e., have true size matching nominal size. In Fig. S6 we compare the observed t-statistic (derived from z-scores computed within matching condition c) with the distribution computed from 999,999 surrogates, while in Table S4 we show the p-values reached with a one-sided hypothesis test from these surrogates and this discriminating statistic. The p-values

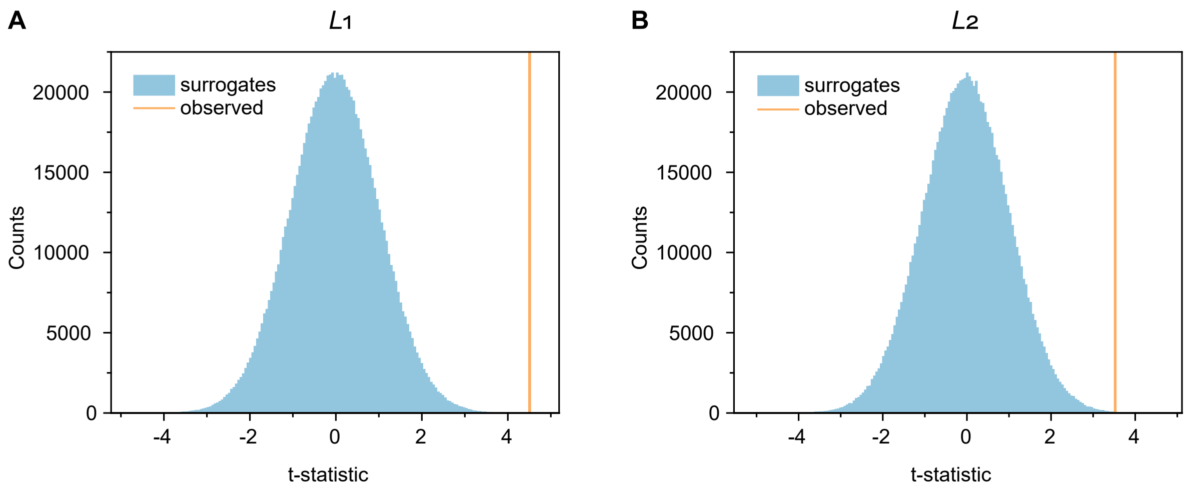

Figure S6: Observed t-statistic (orange vertical line) and histogram of distribution under surrogates (orange) representing hypothesis H_0^i where (A) $i = 1$ and (B) $i = 2$.

reached using rigorous surrogate methods are similar to those attained through the t-test.

VI Compatibility with feed-forward neural networks

To investigate the general applicability of the wiring cost controlled training strategy beyond models with recurrent network structures, we explore its application to multi-layer perceptrons (MLPs). We compare the performance of MLPs trained with rewiring strategies SET, DeepR and DSR on the MNIST, Fashion MNIST, and CIFAR10 datasets (Fig. S7). For the MNIST and Fashion MNIST datasets, the model we use consists of four hidden layers, each containing 256 neurons. The connection density between consecutive layers is set to 0.02 for MNIST and 0.05 for Fashion MNIST. The hidden layer is fully connected to the output layer, and we use a batch size of 128. The training regimen incorporates the Adam optimizer with an initial learning rate of 0.01, and the learning rate is halved every 1/3 of the total training epochs. For the CIFAR10 dataset, we use a three-layer MLP featuring 1500 neurons in each hidden layer. The connection density between consecutive layers (except the hidden layer to the output layer) is set to 0.05. Training is executed using stochastic gradient descent with Nesterov momentum of 0.9, a batch size of 128, initial learning rate of 0.01, and the halving of learning rate at every 1/3 of the total training epochs. The observed trends consistently demonstrate the superior performance of MLPs trained with wiring cost control, suggesting that the integration of wiring cost control presents an opportunity rather than a limiting factor for ANNs.

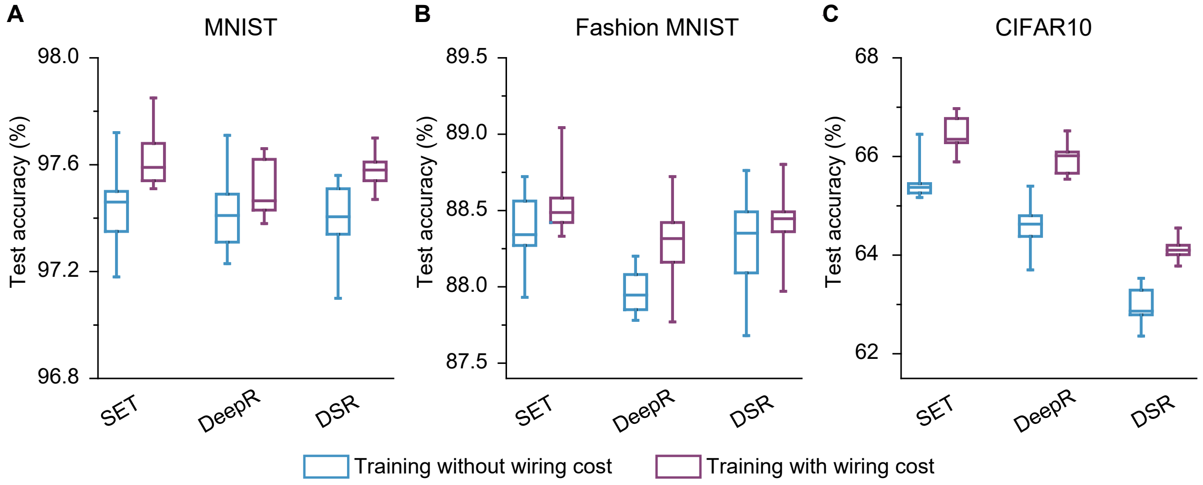

Figure S7: The test accuracy of multi-layer perceptrons (MLPs) trained on (A) MNIST, (B) Fashion MNIST, and (C) CIFAR10 datasets is evaluated. The comparison of performance between models trained without (blue) and with (purple) wiring cost control, employing dynamic sparse rewiring algorithms SET, DeepR and DSR, is conducted. In this study, utilizing the wiring cost controlled framework, we set the wiring cost coefficient η values of 2×10^{-5}, 3×10^{-5}, and 3×10^{-5} for the MNIST, Fashion MNIST, and CIFAR10 datasets, respectively. The boxplots present the minimum, maximum, median, and interquartile ranges (spanning from the 25th to the 75th percentiles) across 10 independent runs.

References

[S1] Valerio Mante, David Sussillo, Krishna V Shenoy, and William T Newsome. Context-dependent computation by recurrent dynamics in prefrontal cortex. *Nature*, 503(7474):78–84, 2013.

[S2] Guangyu Robert Yang, Madhura R Joglekar, H Francis Song, William T Newsome, and

Xiao-Jing Wang. Task representations in neural networks trained to perform many cognitive tasks. *Nature Neurosci.*, 22(2):297–306, 2019.

[S3] Robert Tibshirani. Regression shrinkage and selection via the lasso. *J. R. Stat. Soc. Ser. B*, 58(1):267–288, 1996.

[S4] Arthur E Hoerl and Robert W Kennard. Ridge regression: Biased estimation for nonorthogonal problems. *Technometrics*, 12(1):55–67, 1970.

[S5] Jack Murdoch Moore, Gang Yan, and Eduardo G. Altmann. Nonparametric power-law surrogates. *Phys. Rev. X*, 12:021056, 2022.
